# Optimization of the Transformation Protocol for Increased Efficiency of Genetic Transformation in *Hevea brasiliensis*

**DOI:** 10.3390/plants11081067

**Published:** 2022-04-14

**Authors:** Jinu Udayabhanu, Tiandai Huang, Shichao Xin, Jing Cheng, Yuwei Hua, Huasun Huang

**Affiliations:** 1 Key Laboratory of Biology and Genetic Resources of Rubber Tree, Ministry of Agriculture and Rural Affairs, State Key Laboratory Incubation Base for Cultivation & Physiology of Tropical Crops, Rubber Research Institute, Chinese Academy of Tropical Agricultural Sciences, Haikou 571101, China; jinu.v.bhanu@gmail.com (J.U.); huangtiandai@catas.cn (T.H.); scxin@catas.cn (S.X.); jordanafs@163.com (J.C.); 2 Haikou Key Laboratory of Tropical Plant Seedling Innovation, Haikou 571101, China

**Keywords:** *Hevea* *brasiliensis*, cotyledonary somatic embryos, *Agrobacterium*-mediated transformation, GUS staining, transmission electron microscopy

## Abstract

The recurring growth of bacterium in newly developed resistant cells and a minimal level of bacterial infection rate are the main limiting factors of *Agrobacterium*-mediated transformation experiments in *Hevea brasiliensis*. The current study aimed to optimize crucial factors of the transformation protocol in order to obtain an efficient transformation experimental model for *Hevea* using cotyledonary somatic embryos as explants. Transformation conditions such as antibiotic concentration, preculture duration, *Agrobacterium* concentration, sonication and cocultivation conditions were analyzed using the binary vector pCAMBIA2301. Transient transformation was confirmed by GUS histochemical staining. The best transformation efficiency was observed when the explants were not cultured on a preculture medium that contained acetosyringone at a level of 100 μM. The best results were obtained using a bacterial density of 0.45 at OD 600 nm, 50 s of sonication of explants in a bacterial liquid culture and a total incubation time of 18 min in the same bacterial suspension. Transmission electron microscopical analysis confirmed the impacts of sonication on bacterial infection efficiency. Cocultivation conditions of 22 °C and 84 h of darkness were optimal for the transfer of T-DNA. *Agrobacterium* was eliminated with 500 mg/L of timentin, and the selection of transformants was performed using 100 mg/L of kanamycin in the selection medium. The presence of transgene was confirmed in the resistant embryos by polymerase chain reaction (PCR). The improved method of genetic transformation established in the present study will be useful for the introduction of foreign genes of interest into the *Hevea* genome for the breeding of this economically important plant species in the future.

## 1. Introduction

*Hevea brasiliensis* or the *Hevea* rubber tree, which belongs to the *Euphorbiaceae* species, is the most reliable source for producing natural rubber. It mainly grows in tropical areas. Even though the demand for natural rubber is very high, its production rate is limited due to various challenges, such as tapping panel dryness [1], leaf-fall diseases [2,3], low-yield latex and long juvenile periods [4].

The traditional breeding of *Hevea* is time- and labor-intensive. It can take more than 25 years to recover a new and improved clone of *Hevea* [5]. Different in-vitro tissue culture techniques have already been established to propagate improved clones of *Hevea* [6,7,8]. Somatic embryos (SEs) can be exploited to generate large quantities of propagules in tissue cultures [9,10]. It is possible to achieve an unlimited rate of secondary somatic embryogenesis from a single culture of a *Hevea* primary embryo without creating any genetic instability in the regenerated plantlets [4,11]. The survival rate of somatic *Hevea* seedlings has been found to be remarkable in field trials [11], which is a great success for a woody species and a very promising start for sustained production of the planting material.

To meet the increasing global demands for natural rubber, high throughput propagation methods need to be appropriately combined with the production of improved lines of *Hevea*. Gene editing is the most promising way to overcome limiting factors of latex biosynthesis in *Hevea.* This could be achieved by introducing agronomically important new genes, and also by eliminating some specific genes from the *Hevea* genome [12]. 

In the past few years, many transformation attempts have been performed using *Hevea* for genetic manipulation using in-vitro, as well as in-vivo methods. Even though many transformation methods are available for genetic modification, *Agrobacterium*-mediated transformation is the most efficient method used in *Hevea* [13,14,15,16,17,18]. Transgenic calli were obtained from *Hevea* through infection with *Agrobacterium* strain EHA 105 containing binary vector pCAMBIA2301, which led to high transient glucuronidase (GUS) activity in the callus [15]. Transgenic *Hevea* seedlings were regenerated with the *HbSOD* gene under the control of CaMV 35S promoters [14]. From leaf and root explants, transgenic callus cell lines of *Hevea* were obtained using *Agrobacterium* strain EHA 101 carrying the *MnSOD* gene under the control of the CaMV 35S promoter [19]. All of the previous transformation studies in *Hevea* used embryogenic callus as the explant for the production of transgenic *Hevea* clones. A crucial factor of transformation success is the type of explant with actively dividing cells. Immature anther, inner integument, or leaf-derived embryogenic calli are generally used as successful receptor sources in *Hevea* to obtain transgenic seedlings. Whenever the transformation was conducted using embryogenic calli as the explants in *Hevea*, the transformation efficiency was not satisfactory due to the low level of transgenic seedling regeneration. Recent studies proved that SEs were amenable explants for *Agrobacterium* infection in *Hevea* [11,18]; however, the inability of bacterium to reach the inner cells of the plant tissues was a major drawback [20,21]. Sonication makes micro lesions on the surface and the inner tissues, thus increasing transformation efficiency. The formation of wounds and the presence of acetosyringone during cocultivation at the optimum temperature and duration enabled *Agrobacterium* to actively penetrate the plant tissues and start the T-DNA transfer mechanism in the deeper cells. Sonication-assisted, *Agrobacterium*-mediated transformation (SAAT) has been used in various plant species to achieve a good transformation efficiency [22,23]. 

In the present study, an efficient *Agrobacterium*-mediated transformation method was developed for *Hevea* using SEs as the explants. SEs, at the cotyledonary stage of development, have a high capacity for secondary somatic embryogenesis, which enables the recovery of transgenic plants from genetically transformed cells. The *Hevea* clone Reyan 73397 (Rubber Research Institute (RRI), CATAS, Haikou, China), used in the present study, is the most popular clone and is widely planted in China for the production of all the natural rubber there [4]. Various parameters of transformation were analyzed, and their effect on transformation efficiency was estimated through the transient expression of the GUS reporter gene. This method could be used for the introduction of foreign genes of interest into the same plant species. 

## 2. Results

### 2.1. Kanamycin Sensitivity Analysis

With regard to kanamycin sensitivity, when tested with cotyledonary SEs, it was found that any concentration above 100 mg/L of kanamycin was lethal to the tissues. Although tissue viability was observed at 150 mg/L after a few days, gradual death still occurred (Table 1). It was evident that the lethality was gradual at this concentration with the explant losing its viability after one month. Hence, 100 mg/L of kanamycin was used for the selection of transgenic embryos.

### 2.2. Effect of the Preculture Period on Transformation Efficiency

**Figure 1:**
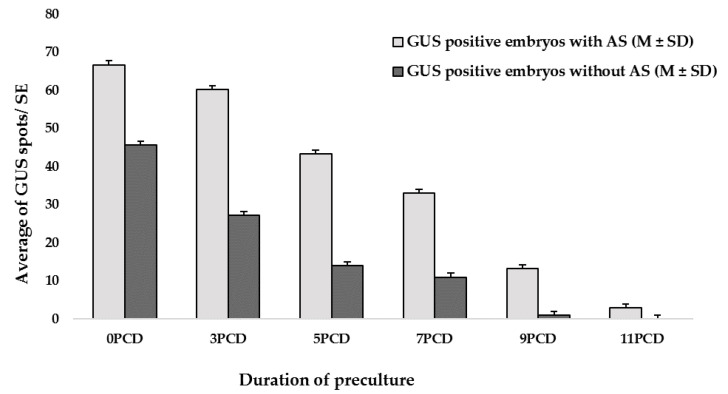
Effect of acetosyringone (AS) and preculture period (0, 3, 5, 7, 9, or 11 preculture days (PCDs)) on the average number of GUS spots per inoculated SE. Number of GUS spots per SE was recorded 3.5 days after bacterial inoculation.

The response obtained from the current study was calculated by analyzing the average of the GUS spots/embryos. The SEs with evident GUS spots were considered as GUS-positive embryos. The number of GUS spots was significantly higher in SEs cultivated on MS - based embryogenesis medium (MSE) [4] supplemented with acetosyringone (AS) than in those cultivated on MSE without AS (Figure 1). The highest transient GUS expression was observed at preculture day zero (0 PCD) of both groups (Figure 1). 3PCD results were also similar to 0 PCD (control). After 3PCD, the morphology of the embryos changed; the abaxial and adaxial sides became harder. The embryo’s color also changed to a pale white color, which showed the maturation of the cotyledons. Therefore, precultivation of SEs on MSE supplemented with AS generally had a negative impact on transformation efficiency. 

### 2.3. Effect of Agrobacterial Concentration on Transient GUS Expression in Explants

**Figure 2:**
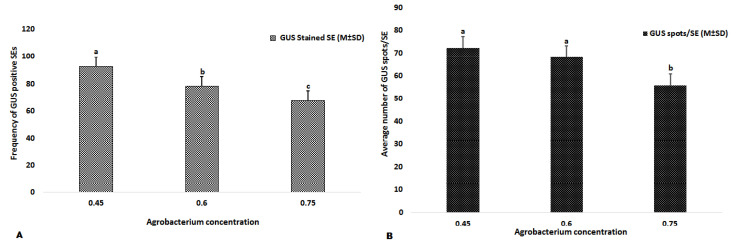
The effect of *Agrobacterium* concentration on frequency of transiently transformed SEs (**A**) and average number of GUS spots/SE (**B**) in *Hevea*. Results are expressed as mean (M) ± standard deviation (SD). The experiments were repeated three times. Means with the same letter above the bars were not significantly different at the 0.05 level according to Duncan’s *t* test. The SEs with deep GUS staining were termed ‘GUS-stained SE’.

To determine the optimal concentration of *Agrobacterium* for inoculating the SEs, bacterial suspensions at three different optical densities, OD600 0.45, 0.6 and 0.75 were tested. The explants (SEs) were stained after the cocultivation period to analyze the transient GUS expression. The rate of GUS transient expression in the SEs was significantly higher when using the concentration of OD600 = 0.45 than OD600 = 0.75, and only slightly higher than when using OD600 = 0.6 (Figure 2). Therefore, OD600 = 0.45 was confirmed as the optimal concentration of *Agrobacterium* for inoculating SEs.

### 2.4. Sonication-Assisted, Agrobacterium-Mediated Transformation

**Figure 3:**
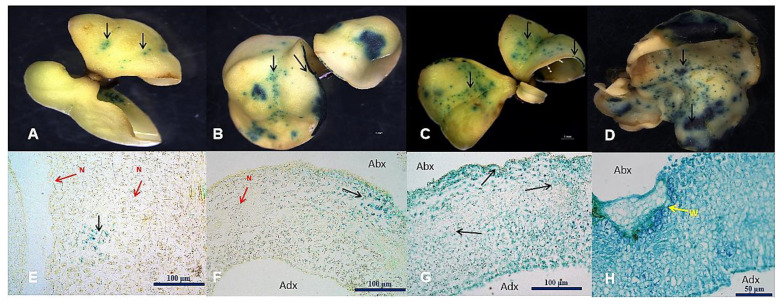
Various levels of GUS expression in *Hevea brasiliensis* after sonication frequency analysis. (**A**,**E**) 8 min of agrobacterial inoculation with 10 s sonication, black arrows indicate GUS expression in the vascular bundles and red arrows (N) indicate no transformed areas; (**B**,**F**) 8 min of infection with 30 s sonication, black arrows indicate GUS signals in the epidermis and vascular bundles in the explant; (**C**,**G**) show strong GUS signaling after 50 s sonication and 18 min of infection; the histogram in (**G**) clearly indicates the GUS signals in the epidermis and all the deeper cells inside the tissues, GUS signals were present in the abaxial (Abx) and adaxial (Adx) regions; (**D**,**H**) show 70 s sonication with 18 min of inoculation and lethal micro wounds (yellow arrow marked W) for the survival of tissues for secondary embryogenesis; GUS stain is spread all over the tissues in histogram (**H**). Bars are 50 μm and 100 μm.

Different sonication durations were used with the *Agrobacterium* culture to inoculate the SEs. Various levels of GUS expression and morphological changes in the SEs were observed in each treatment. Anatomical observation confirmed that the tissue (Figure 3) was lethally affected if the duration of sonication was above 50 s. After several trials, the best infection mode was 8 min of incubation of the SEs in the bacterial suspension culture, then 50 s sonication, followed by another 10 min of incubation in the same liquid culture without shaking.

### 2.5. Screening of Cocultivation Condition

Based on the GUS-positive results obtained from the present study, it was understood that the level of GUS expression varied according to the cocultivation condition. In higher temperature groups (25 and 28 °C), the intensity of GUS staining was high, and it was difficult to identify the spots. In addition, bacterial growth was very high around the SEs in these temperatures. The appearance of blue spots was highly evident in the minimum temperature group (22 °C), and the bacterial growth was the lowest in this group (Table 2). Hence, based on the results, 22 °C was fixed as the optimum temperature for the cocultivation of SEs.

To obtain stronger GUS signaling in the SEs without the recurring growth of bacteria, the cocultivation period was checked at 22 °C. It was clear from the results that the cocultivation duration played a very crucial role in GUS expression, and also in bacterial growth. The highest frequencies (%) of SEs with GUS expression (0.66 ± 0.03, 0.73 ± 0.03) were observed in the 84 h and 90 h groups, respectively; however, the highest number of GUS spots per SE (88.67 ± 3.28) was achieved in the 84 h cocultivation group. The other two groups (72 and 78 h) showed the minimum and a moderate level of GUS expression. Based on the results, the cocultivation period was confirmed at 22 °C with a duration of 84 h in dark conditions (Table 3). 

### 2.6. GUS Histological Observation

**Figure 4:**
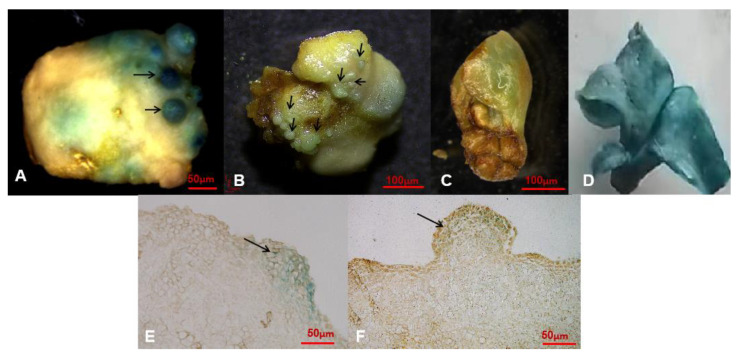
Secondary somatic embryos expressing GUS gene. (**A**) Eighteen days culture with evident GUS-carrying globular embryo development; (**B**) twenty-five days culture with GUS-positive globular embryos under a kanamycin selection pressure of 100 mg/L; (**C**) cotyledonary secondary somatic embryos from 45-day-old cultures; (**D**) GUS expression in transgenic SEs; (**E**) a cross section of secondary embryos with GUS signals, 14-day-old cultures; (**F**) secondary globular embryo showing GUS signals, 18-day-old cultures. Bars are 50 μm and 100 μm. Black arrows indicate GUS expressions.

Positive transgenics showed a blue color after 8–10 h incubation in GUS staining solution at 37 °C. Anatomical observation of infection localization in positive GUS-stained plant tissues was performed under a light microscope. Stable GUS expression was confirmed in positive resistant embryos (Figure 4A–D). Highly promising GUS signals were spread all over the cells inside the resistant embryos (Figure 4E,F).

### 2.7. Transmission Electron Microscopy 

**Figure 5:**
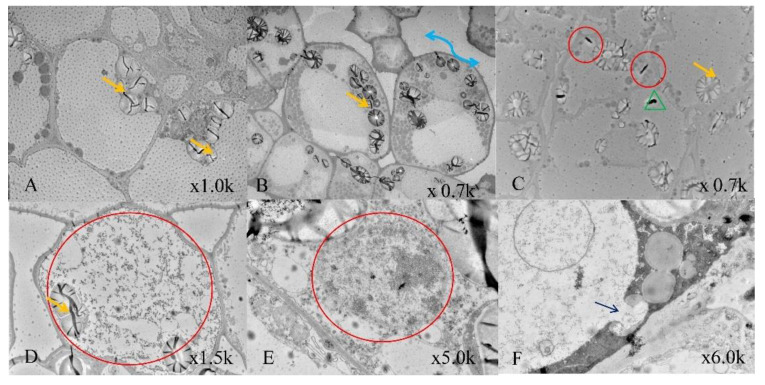
TEM analysis of cells of *Agrobacterium*-inoculated somatic embryos. (**A**) Control cells with normal internal structures, the cell wall and starch granules (yellow arrows) were evident and normal; (**B**) sonication effect on cellular structure, the intercellular space is disturbed (blue mark) and the arrangements of starch granules (yellow arrows) was also affected; (**C**) cells with normal agrobacterial infection showing very few numbers of plasmids (red circles) inside the cell wall, green marks indicate mitochondrion; (**D**,**E**) 50 s sonication and 18 min agrobacterial inoculation showing the bulk of plasmids inside the cells; (**F**) 70 s sonication and 18 min agrobacterial inoculation showing the lethality in the cell wall, evident breakage (navy blue arrows) is present in the cell wall, and all cellular components are shown to have leaked out.

Transmission electron microscopy (TEM) observation clearly indicated the anatomical changes in each test group (Figure 5). The non-transformed control cells showed very normal anatomies. Well-organized cell walls, cytoplasm and cell organelles were observed. Numerous amyloplasts with starch grains were highly noticeable in all the samples. Various cytoplasmic components, such as mitochondria, nucleus, trichome and vascular bundles, were regularly present in all samples. In the second group (50 s sonication at 40 kHz), intercellular space between the cells was increased. The starch granule arrangements were also altered inside the cytoplasm due to the sonication; however, in samples after 18 min of *Agrobacterium* inoculation, a normal anatomy could be observed that was similar to the control but with lower levels of bacterial presence. However, the samples with 50 s sonication and 18 min inoculation had evident bacterial colonization in and around the cell walls. The higher sonication (70 s) and 18 min *Agrobacterium* inoculation showed very lethal wounds in the cell walls and leakage of cellular organelles out of the cytoplasm.

### 2.8. Molecular Confirmation of Genetic Transformation

**Figure 6:**
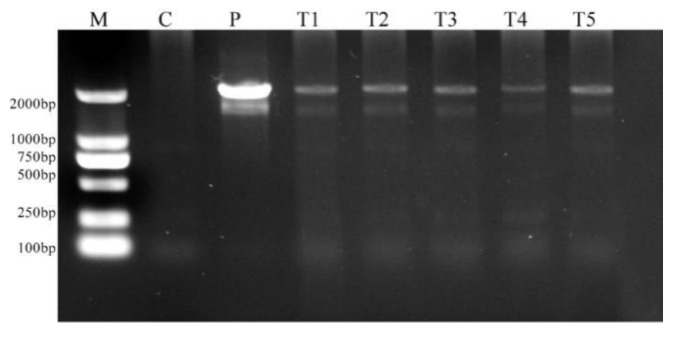
PCR amplification of transgene (GUS) in the resistant secondary somatic embryos obtained from the explants after being cultured for 40 days in the selection medium. M: DNA marker; C: negative control; P: positive control, plasmid DNA; T1–T5: kanamycin-resistant embryos.

Genomic DNA was isolated from putatively transformed kanamycin-resistant embryos from the SE explants in the selection medium. It was subjected to PCR amplification and compared with DNA from untransformed SEs (negative control). The presence of the GUS gene was confirmed by the amplification of 2000 bp fragments in the resistant embryos (Figure 6). The bands were compared with the positive control plasmid vector pCAMBIA2301 and DL2000 DNA marker (Takara, Kyoto, Japan). No bands were observed in the negative control. 

## 3. Discussion

In *Hevea*, success of *Agrobacterium*-mediated transformation and further development of transgenic tissues and plantlet regeneration are greatly dependent on the nature of the explant and the experimental procedure. In this study, SAAT was successfully carried out using cotyledonary SEs as target plant tissues for the transformation experiment. The infected SEs were recovered without bacterial overgrowth, and later, transgenic secondary somatic embryos were obtained.

*Hevea* somatic seedling development from a callus culture is a very complicated developmental process. A repeated subculture of callus may lead to mutations and epigenetic changes [8]. To overcome these problems, SEs can be used as explants. The cyclic development of embryos is possible when SEs are explants [4]. Reports are available about the successful system for secondary somatic embryogenesis using clones of *Hevea* [4]. The expression of various transgenes *(uidA* and *nptII*) was comparably stable in transgenic clones of *Hevea* after three successive buddings [24,25], yet a noticeably lower level of GUS activity and higher somaclonal variation occurred in the later generations of budded plants, compared with self-rooting transgenic clones [26]. The resistant embryo generation progressively increased in *Hevea* during repeated proliferation cycles [18]. This signified the potential of somatic embryogenesis for the propagation of transgenic plants; thus, we selected SEs as explants for bacterial infection and for secondary embryogenesis after the transformation experiment. 

After analyzing various reports, it was very clear that the success rate of *Agrobacterium* transformation varied according to the explant type, even in the same species [19]. The other factors involved in *Agrobacterium* transformation efficiency, such as selection agent (kanamycin), mode of infection, preculture and cocultivation conditions, also changed depending on the nature of explant. 

A measure of 300 mg/L of kanamycin was used for selecting transformed cell lines in *Hevea* from immature anther, derived calli and a 4% transformation efficiency was obtained [14]. This high concentration helped to prevent escapes from occurring. In another study 50 mg/L of kanamycin was the optimum concentration for obtaining the maximum transformation efficiency in different explants, such as the leaves and roots of *Hevea* [19]. In dicotyledonous species, including *Hevea*, kanamycin is usually used as a suitable selection agent to screen transgenic cell lines. A measure of 100 mg/L of kanamycin is effective in *Hevea,* without causing too much necrosis, to stop the growth of untransformed anther callus (escapes), and allows the proliferation of transformed callus [13]. A measure of 100 mg/L of kanamycin, along with 40 mg/L hygromycin and 200 mg/L cefotaxime, was used to improve transformation efficiency [27] using *Agrobacterium* in a legume species (*Trifolium subterraneum* L.). Kanamycin selection efficiency has been well proven [28] for developing the transgenic biofuel plant *Jatropha curcas*. Based on the type of explant chosen, the kanamycin concentration also differed in the same species [13,14,24]. In the current study, transformed cell lines were obtained in MSE containing a 100 mg/L concentration of kanamycin through secondary somatic embryogenesis using cotyledonary SEs as explants. 

Preculture treatments are promising for transformation experiments in various plants [29,30]; however, preculture treatment did not show any remarkable effect in kiwi fruits and *Eucalyptus saligna* for enhancing transformation efficiency [31,32]. Based on the current findings, it could be understood that preculturing was not essential for the transformation method when using SEs as explants.

When the *Agrobacterium* concentration used to inoculate the SEs was very high, it was difficult to wash out the *Agrobacterium* from the explants after cocultivation, and would lead to frequent *Agrobacterium* contamination. Therefore, it was better to choose a relatively low concentration with a high infection ability [33]. In this study, we found that OD600 = 0.45 was the optimum concentration for the T-DNA transfer mechanism using the current transformation protocol. Transformation was carried out using the sonication technique to obtain efficient *Agrobacterium* infection. According to various research reports, sonication is the best choice for the enhancement of transformation efficiency, with optimum expression of a foreign gene into the host DNA [23,34,35]. A 40 kHz frequency and less than 1 min sonication could be a supporting factor for *Agrobacterium* infection in the SEs of *Hevea*. Cocultivation period was the most crucial time for the enhanced expression of transgenes. According to various studies in different plant species [36,37,38], the cocultivation duration and temperature has a great significance in the success of *Agrobacterium*-mediated transformation. The current result compromised the findings of various researchers who performed a similar study [39,40,41]. 

The *Agrobacterium*-mediated transformation study in embryogenic calli of the oil palm tree showed 25.72% GUS expression in a cocultivation period of 5 days [36]. In another transformation study that was performed with a medicinal orchid plant (*Dendrobium lasianthera*), the highest transformation efficiency was obtained with a cocultivation period of 5 days at a rate of 65% [38]. A recent study claimed that the duration of the cocultivation had a significant effect on transient GUS expression [42]. The authors proved that a longer cocultivation period (more than 3 days) might lead to the browning of the explants, and a decreased level of GUS expression in commercial hybrid passion fruit KPF4. Another study claimed that dropping the temperature to 20 °C and expanding the callus cocultivation period (5–7days) greatly increased the transformation frequency in the *Hevea* callus lines [15]. The mechanism behind the temperature sensitivity of the process of genetic transformation has been well known for years. T-pilus formation is a highly temperature-dependent process, since some of its components, such as VirB3 and VirD4, have only been detected at 20 °C, and thus the highest amounts of T pili were detected at 20 °C [43]. In this study, bacterial proliferation was diminished at 22 °C during cocultivation without browning in the SEs. Despite this, the bacterial proliferation was higher after 90 h cocultivation; thus, the present study confirmed that a cocultivation period of 84 h (3.5 days) at 22 °C was optimal for obtaining the highest GUS expression with the modified SAAT method in *Hevea*.

In the present study, we observed that the development of resistant secondary embryos occurred mainly in the epidermal cells. Earlier studies reported that SSE-derived epidermal cells originated from a single cell [11,18], and microscopic analysis of transient GUS expression confirmed *Agrobacterium* infection in both epidermal and subepidermal cells [18]. This strengthened our observation of the formation of resistant SSEs and signified the stable expression of GUS. Nevertheless, chimeric occurrences need to be verified in further experiments. 

Cytoplasmic organelles and anatomical changes under the effect of sonication coupled transformation could be analyzed using TEM, based on recent studies [44,45]. The infection rate was very impressive in *Agrobacterium*-mediated transformation with sonication (50 s, 40 kHz). This might be the reason behind the formation of more transgenic resistant embryos after 16–20 days of culturing. After 16 days post-transformation, secondary somatic embryogenesis could be observed. The resistant embryos developed into torpedo and the later cotyledon stage SEs during the 30 days that followed. GUS staining and PCR analysis confirmed the GUS gene integration in the secondary somatic embryos that were raised from the SEs after transformation. 

## 4. Materials and Methods

### 4.1. Selection of Explant

Mature cotyledonary SEs of *Hevea* (Reyan 73397), 1.5 cm in size, were selected from in vitro-grown cultures, which were provided by the Innovation Base for production of Natural Rubber New Planting Material, Danzhou, China. The embryos were then cultured on a Murashige and Skoog (MS) [46]-based embryogenesis medium (MSE) [4] with a slight modification (4.44 μM 6-benzyladenine, 13.9 μM Kinetin (KT),1.44 μM Gibberellic acid, 0.27 μM 2,4 D 2,4-Dichlorophenoxyacetic acid (2,4-D), 70 g/L sucrose, 50 ml coconut water, 1 g charcoal and 2.2 g/L phytagel) in a 100 mm × 20 mm Petri dish (Sigma, St. Louis, MO, USA).

### 4.2. Kanamycin Sensitivity Analysis

SEs were used as the target material for *Agrobacterium* infection. To determine a suitable concentration for the selection of transgenic cells, the sensitivity of the SEs was tested using different concentrations of kanamycin. The SEs were propagated in a selection medium, MSE supplemented with 0, 50, 75, 100, 125 and 150 mg/L kanamycin. Three replicates with 30 SEs in total were used per treatment. After 5 weeks, the developed SSEs were counted and expressed as the mean number per SE used.

### 4.3. Screening Effect of Preculture Days (PCD)

SEs were precultured for 0–11 days before they were transformed on a preculture medium (PCM, MSE without or with 100 μM acetosyringone). The cultures were maintained in dark conditions at 24 °C. The experiment was repeated for three batches of 10 SEs/treatment. The number of GUS spots per SE was recorded 3.5 days after bacterial inoculation, and the mean number of GUS spots per inoculated SE was presented.

### 4.4. Preparation of Agrobacterium Strain 

**Figure 7:**
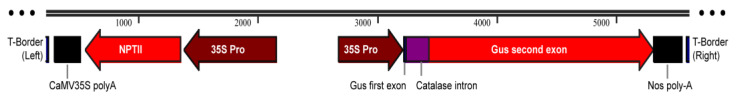
Schematic representation of T-DNA from pCAMBIA2301 showing the neomycin-phosphotransferase II (*nptII*) and GUS reporter gene (*uidA*) driven by the constitutive 35S promoter (35S Pro).

The *Agrobacterium tumefaciens* strain EHA 105 [47], carrying binary vector pCAMBIA 2301 (Cambia, Canberra, Australia), was used for genetic transformation. T-DNA of the binary vector contained a selectable marker gene encoding neomycin-phosphotransferase II (*nptII*), conferring resistance to kanamycin, driven by the CamV35S promoter (Figure 7). The bacteria were initially cultured on a Luria-Bertani agar (LBA) plate containing 100 mg/L of kanamycin and incubated at 28 °C for three days. A single colony was obtained from the LBA plate and inoculated in 50 ml LB broth containing 100 mg/L of kanamycin, followed by incubation at 28 °C at 220 rpm for 12 h. The culture was centrifuged at 4300× *g* (25 °C) for 10 min and the pellet was resuspended in MS resuspension medium (MSRM, MSE with sucrose 3.5%) until the OD600 nm reached 0.45, 0.6 and 0.75. Then, 100 µM acetosyringone was added to the bacterial cell suspensions, which were then incubated again at 28 °C in 220 rpm for 4 h.

### 4.5. Sonication-Assisted, Agrobacterium-Mediated Transformation (SAAT) 

After 4 h incubation in a shaker, bacterial cultures were used to inoculate the SEs. This experiment was framed out with modified parameters based on a previous study [23]. Various sonication (10, 30, 50 and 70 s) and inoculation (8, 18 min) durations were used in this experiment. Experimental protocols were as follows: 8 min of SE inoculation, followed by 10, 30, 50 and 70 s of sonication; 18 min of SE inoculation followed by 10, 30, 50, or 70 s of sonication. Sonication was performed by placing the culture flask containing the explants in the middle of the sonicator (40 kHz), and it was then kept in laminar airflow to dry the SEs using sterilized filter paper. After blot drying, it was placed on PCM for cocultivation in the dark at 22 °C. The efficiency of GUS expression was assessed by calculating the average number of GUS spots per SE. 

### 4.6. Screening of Cocultivation Condition

After *Agrobacterium* inoculation, the SEs were grouped for evaluating the effect of the coculture conditions for optimum GUS expression, as well as to avoid the occurrence of bacterial growth. To prevent bacterial growth in the explants, different temperatures, such as 22 °C, 25 °C and 28 °C, were examined during a 4-day cocultivation. 

In order to understand the impacts of cocultivation duration on GUS expression, the results were checked periodically at 72, 78, 84 and 90 h, respectively. The inoculated SEs were placed in coculture medium (PCM, MSE with 100 µM acetosyringone) and co-cultivated in the dark at 22 °C. Each set of experiments were repeated three times with 10 SEs per treatment. 

### 4.7. Histochemical GUS Enzyme Assay

After cocultivation, the SEs were examined for transient GUS expression [48]. GUS-positive SEs were washed 4–5 times with 70% ethanol in order to improve the visualization of the infection sites under the light microscope.

### 4.8. Transmission Electron Microscopy 

The co-cultivated SE samples were used for transmission electron microscopy (TEM) analysis. The samples were cut into small pieces (2 mm × 2 mm) and immediately immersed in a mixture of 3% glutaraldehyde in a 0.2 M sodium-cacodylate buffer (pH 7.4) and incubated for 3–4 h at 4 °C. This chemical mixture acted as a fixative solution. Later, the fixative solution was removed from the sample by repeated rinsing in a diluted sodium-cacodylate buffer for 30 min at 4 °C. Post-fixation of samples was then performed using 2% osmium tetroxide (OsO4) in cacodylate buffer for 1 h at room temperature. The dehydration process was completed using a graded ethanol series [70% (3 × 15 min), 95% (3 × 15 min) and 100% (2 × 30 min)] and propylene oxide (2 × 30 min). Eventually, the pieces were embedded in Epox resin and allowed to completely polymerize. The resin blocks were then cut into semi-thin sections using an ultra-microtome set to a thickness of 1 μm and stained with toluidine blue and methylene blue for optical observations. Ultrathin sections (70 nm) were double stained with saturated uranyl acetate and lead citrate. Observation and detailed documentation of each sample were performed using the HT7700 TEM system (Hitachi, Tokyo, Japan) at 80 kV.

### 4.9. Selection of Transgenic-Resistant Secondary Somatic Embryos

After cocultivation, the inoculated SEs were placed on MSE for two days without any antibiotics. They were then cut into small pieces and placed on an MS-based callus induction medium (3.0 mM Calcium chloride, 7.0 µM KT, 8.1 µM Naphthalene acetic acid, 6.8 µM 2,4-D, 204.5 mM sucrose and 2.2 g/L phytagel) containing different concentrations of kanamycin (25, 50, 75, 100, 125 and 150 mg/L) and 500 mg/L of timentin and kept in dark conditions at 24 °C. After 16 days, the formation of globular embryos was noticed, and they were transferred to MSE containing the same selection agents. The developed resistant embryos were taken for transgene confirmation and somatic seedling development.

### 4.10. Molecular Confirmation of Transgenic Gene

Genomic integration of the GUS gene in kanamycin-resistant embryos was confirmed by PCR analysis. Non-transformed embryos were used as a negative control, and plasmid DNA as a positive control. The genomic DNA was isolated using a commercial kit (TransGene Biotech, Beijing, China). The PCR reactions were carried out using the S1000 Thermo cycler (Bio-Rad, Hercules, CA, USA). The PCR reaction mixture (25 µL) containing 100 ng of DNA, 2.5 μl of PCR buffer (10×), 200 μM deoxyribose nucleotide triphosphate (dNTP), 2.5 mM MgCl2, 200 nM of forward and reverse primers and 1U Taq polymerase (R001B Takara, version No. R045A). The amplification protocol was: initial denaturation at 98 °C for 5 min, then denaturation at 98 °C for 30 s, followed by 30 s of annealing at 56 °C and extension at 72 °C for 1 min in 35 cycles, followed by the final extension at 72 °C for 10 min. Amplified DNA was electrophoresed in 1% agarose gels, stained with ethidium bromide, and the amplified DNA bands were visualized under an Ultra-violet transilluminator and documented.

### 4.11. Statistical Analysis

In the antibiotic selection experiment, the frequency of chlorosis and bleaching was calculated by dividing the number of damaged embryos by the total number of embryos used and secondary somatic embryogenesis was calculated by finding the mean number of secondary somatic embryos (SSEs) per primary SE. The frequencies of GUS-positive embryos were calculated by taking the number of GUS-stained SEs and dividing them by the total number of embryos ×100 and presenting an average number of GUS spots per SE. Duncan’s multiple range test (*p* < 0.05) was carried out with the Graph Pad Prism 5 software.

## 5. Conclusions

The current study emphasized the efficiency of the modified transformation system in SEs of *Hevea*. We optimized the crucial factors of transformation. No preculture days were required for this experiment, and a minimum temperature of 22 °C favored the cocultivation period (84 h) for excellent GUS expression. Resistant transgenic secondary embryos could be observed from the explants within 20 days post-transformation. PCR analysis detected the presence of the GUS gene (*uidA*) in the resistant embryos. It also supported the use of secondary embryogenesis for the propagation of transformed lines. We could achieve torpedo-shaped resistant embryos 40 days after transformation. Even though we achieved optimum transformation conditions for the finest formation of transgenic SSEs, the occurrence of escapes was a risk factor for the success rate of transformation. The mechanisms behind the escapes and antibiotic dose, as well as its influence on the secondary somatic embryogenesis, need to be investigated using the same explant. The developing transgenic seedlings will be screened to determine their survival rates, and a genetic analysis will be performed to understand the effect of transgene expression on the incidence of growth.

## Figures and Tables

**Table 1:** The frequency of chlorosis and mean number of secondary somatic embryos (SSEs) per primary SE in the selection medium with different concentrations of kanamycin after 40 days of culture.

Treatment	Explant	Chlorosis and Bleaching	SSE
Control	30	0	110 ± 6.43
25	30	0 ± 0.58	97 ± 7.00
50	30	16 ± 1.00	74 ± 6.23
75	30	22 ± 3.21	65 ± 3.51
100	30	39 ± 2.51	42 ± 4.72
125	30	67 ± 0.05	14 ± 3.05
150	30	78 ± 1.15	1 ± 1.00

SSE: secondary somatic embryos. Results are expressed as mean (M) ± standard deviation (SD). The experiments were repeated three times.

**Table 2:** The effect of cocultivation temperature on GUS expression in SEs.

Temperature	Duration	Number of Replicates	Average of GUS-Positive Embryos	Average GUS Spots/Embryo
22 °C	4 days	3	0.53 ± 0.03 ^a^	24.90 ± 1.45 ^a^
25 °C	4 days	3	0.43 ± 0.03 ^b^	10.96 ± 1.78 ^b^
28 °C	4 days	3	0.43 ± 0.03 ^b^	7.93 ± 2.49 ^b^

Values are expressed as mean ± standard deviation of the number of GUS-positive SEs and average GUS spots per SE obtained after three replicates of the experiment. Means denoted by the same letter do not significantly differ at the 0.05 level according to Duncan’s *t* test.

**Table 3:** Screening of cocultivation time for GUS expression below 22 °C.

Time (h)	Temperature	No. of Replicates	GUS-Expressed SEs	GUS Spots/Embryos
72	22 °C	3	0.33 ± 0.03 ^c^	27.33 ± 4.63 ^c^
78	22 °C	3	0.53 ± 0.03 ^b^	60.67 ± 6.33 ^b^
84	22 °C	3	0.6 ± 0.03 ^a^	88.67 ± 3.28 ^a^
90	22 °C	3	0.73 ± 0.03 ^a^	79.67 ± 2.33 ^a^

The duration effect on the transformation effect is expressed as mean ± standard deviation of the number of GUS-positive SEs and the average number of GUS spots per SE obtained after three replicates. Means represented by different letters in each column show significant difference at 0.05 level according to Duncan’s *t* test.

## Data Availability

All related data are available within the manuscript, and further information could be obtained on request from the corresponding authors.
